# Gut Microbiota Composition and Fecal Metabolic Profiling in Patients With Diabetic Retinopathy

**DOI:** 10.3389/fcell.2021.732204

**Published:** 2021-10-15

**Authors:** Zixi Zhou, Zheng Zheng, Xiaojing Xiong, Xu Chen, Jingying Peng, Hao Yao, Jiaxin Pu, Qingwei Chen, Minming Zheng

**Affiliations:** The Second Affiliated Hospital of Chongqing Medical University, Chongqing, China

**Keywords:** diabetic retinopathy, gut microbiota, fecal metabolic phenotype, metabolomics, correlation analysis

## Abstract

Recent evidence suggests there is a link between metabolic diseases and gut microbiota. To investigate the gut microbiota composition and fecal metabolic phenotype in diabetic retinopathy (DR) patients. DNA was extracted from 50 fecal samples (21 individuals with type 2 diabetes mellitus-associated retinopathy (DR), 14 with type 2 diabetes mellitus but without retinopathy (DM) and 15 sex- and age-matched healthy controls) and then sequenced by high-throughput 16S rDNA analysis. Liquid chromatography mass spectrometry (LC-MS)-based metabolomics was simultaneously performed on the samples. A significant difference in the gut microbiota composition was observed between the DR and healthy groups and between the DR and DM groups. At the genus level, *Faecalibacterium*, *Roseburia*, *Lachnospira* and *Romboutsia* were enriched in DR patients compared to healthy individuals, while *Akkermansia* was depleted. Compared to those in the DM patient group, five genera, including *Prevotella*, were enriched, and *Bacillus*, *Veillonella*, and *Pantoea* were depleted in DR patients. Fecal metabolites in DR patients significantly differed from those in the healthy population and DM patients. The levels of carnosine, succinate, nicotinic acid and niacinamide were significantly lower in DR patients than in healthy controls. Compared to those in DM patients, nine metabolites were enriched, and six were depleted in DR patients. KEGG annotation revealed 17 pathways with differentially abundant metabolites between DR patients and healthy controls, and only two pathways with differentially abundant metabolites were identified between DR and DM patients, namely, the arginine-proline and α-linolenic acid metabolic pathways. In a correlation analysis, armillaramide was found to be negatively associated with *Prevotella* and *Subdoligranulum* and positively associated with *Bacillus*. Traumatic acid was negatively correlated with *Bacillus*. Our study identified differential gut microbiota compositions and characteristic fecal metabolic phenotypes in DR patients compared with those in the healthy population and DM patients. Additionally, the gut microbiota composition and fecal metabolic phenotype were relevant. We speculated that the gut microbiota in DR patients may cause alterations in fecal metabolites, which may contribute to disease progression, providing a new direction for understanding DR.

## Introduction

Diabetic retinopathy (DR) is one of the most common complications of diabetes mellitus and leads to vision-threatening damage to the retina, eventually leading to blindness. It was estimated that the number of people with DR would increase globally from 126.6 million in 2010 to 191.0 million by 2030. If urgent action was not taken, the number with vision-threatening diabetic retinopathy (VTDR) would increase from 37.3 to 56.3 million (Zheng et al., 2012). DR is a vascular and neurodegenerative disease with complex pathogenesis and progression that is mainly characterized by recurrent episodes of capillary occlusion and progressive local retinal ischemia. Previous studies have revealed that activated CCR5 + CD11b + mononuclear macrophages were involved in early DR (Serra et al., 2012). NLR family pyrin domain containing 3 (NLRP3) inflammasome disorder might cause diabetic retinal damage and destruction via the proinflammatory cytokines IL-1β and IL-18 (Raman and Matsubara, 2020). P2 × 7R, a member of the P2XR family of ATP-gated plasma membrane receptors, has been verified to regulate inflammatory and immune responses. P2 × 7R stimulation or overexpression triggered VEGF secretion and promoted diabetic retinopathy (Raman and Matsubara, 2020). The above mentioned studies suggested that an abnormal immune response and the release of inflammatory factors may play an important role in DR progression. Notably, the human gut microbiota and its effects on the metabolic phenotypes have been shown to play a critical role in the maintenance of immune homeostasis (Scher et al., 2015) and anti-inflammation (Al Bander et al., 2020).

The intestinal microbiota played an important role in the metabolic health of the human host and was implicated in the pathogenesis of many common metabolic diseases, including obesity, type 2 diabetes and non-alcoholic liver disease (Fan and Pedersen, 2021). Studies have shown that people with type 2 diabetes mellitus (T2DM) have malnutrition-associated changes in their gut microbiota (Qin et al., 2012; Karlsson et al., 2013). Previous animal studies have found that intermittent fasting-mediated changes could prevent DR by restructuring the microbiota toward species producing taurochenodeoxycholate (TUDCA) and subsequent retinal protection by TGR5 activation (Beli et al., 2018). Therefore, TGR5, the TUDCA receptor, could be a new therapeutic target for DR, which suggested that gut microbial changes may be associated with DR. Besides, dysbiosis occurs in the gut microbiota of people with T2DM and DR more frequently than in healthy individuals, and the interaction of fungal genera differs between them (Jayasudha et al., 2020).

Metabolomics is based on genomics, transcriptomics and proteomics to identify and quantify low-molecular-weight metabolites in biological samples, thus revealing physiological changes influenced by external stimuli or interventions. An abundance of studies applying metabolomic approaches have been used to identify specific metabolic phenotypes in intraocular fluid (vitreous humor, aqueous humor) and blood samples (Jin et al., 2019; Zhu et al., 2019; Wang H. et al., 2020). However, to our knowledge, few metabolomic studies have investigated fecal metabolic phenotypes in DR. To identify the role of the microbiota and metabolites in DR progression, we analyzed the gut microbiota composition and the fecal metabolic phenotype in the study groups using 16S rDNA sequencing and LC-MS-based metabolomics. Our study explored the composition of the gut microbiota and its associated fecal metabolic phenotype in patients with DR. We hypothesized that the gut microbiota of DR patients may lead to altered fecal metabolites, which may contribute to disease progression.

## Materials and Methods

### Study Participants and Sample Collection

The study included 50 individuals who were enrolled from the Second Affiliated Hospital of Chongqing Medical University (Chongqing, China) from December 2019 to December 2020. 50 individuals were divided into three groups: 21 T2DM DR patients (14 men and 7 women), 14 T2DM DR patients with type 2 DM only, and 15 healthy controls. The three groups were closely matched in terms of age (59.57 ± 9.09, 56.13 ± 8.88, 61.93 ± 6.20). The study received the approval of the Ethics Committee of the Second Affiliated Hospital of Chongqing Medical University (2019(012)) and all participants signed informed consents. All procedures in this study followed the Declaration of Helsinki. All 21 subjects met the following inclusion criteria: (a) patients with DR diagnosed by previous slit-lamp biomicroscopy and fluorescein angiography examinations; and (b) patients without other eye diseases or systemic diseases with ocular complications, such as glaucoma, uveitis, ocular trauma, and age-related macular degeneration. All 14 T2DM patients met the following inclusion criteria: (a) all met the 2018 American Diabetes Association Medical Diagnostic Criteria for Diabetes (American Diabetes Association, 2018); and (b) diabetic retinopathy was ruled out by fundus photography and ocular optical coherence tomograph (OCT) examination. The exclusion criteria for each group were as follows: recent treatment with probiotics, antibiotics, or corticosteroids; gastrointestinal tract surgery (<1 month prior to sample collection); a history of autoimmune diseases including rheumatoid arthritis, psoriatic arthritis, systemic lupus erythematosus and inflammatory bowel disease; type 1 diabetes or unclear etiology of diabetes; and hypertension, obesity, malignant tumors or a history of organ transplantation.

DR and DM patients took metformin with or without insulin injections for glycemic control (duration >3 months). Individuals enrolled in our study had a normal diet and regular bowel movements.

Morning fecal samples were collected after defecation at hospital. Then stool samples were placed into two cryotubes and immediately transported on dry ice within 10 min. All samples were collected by a designated doctor. Fecal samples were stored at −80°C until processing.

### Fecal DNA Extraction and 16S Sequencing

According to the manufacturer’s recommendation, microbial DNA was extracted from fecal samples using a Power Soil DNA Isolation Kit (MoBio Laboratories). Total genomic DNA from samples was extracted using the CTAB method, and the DNA quality and quantity were assessed by the ratios of 260 nm/280 nm and 260 nm/230 nm. The ratios ranged from 1.7 to 1.9 of 260 nm/280 nm and exceed 2.0 of 260 nm/230 nm were considered good. The 16S rRNA V3-V4 region was amplified using the specific primers 341F (CCTACGGGRSGCAGCAG) and 806R (GGACTACV VGGGTATCTAATC). PCR amplification was conducted in a total volume of 50 μl, which included 0.2 μl Q5 High-Fidelity DNA Polymerase, 10 μl Buffer, 1 μl dNTP, 10 μl High GC Enhancer, 10 μM of each primer and 60 ng genome DNA. Thermal cycling conditions were performed as follows: an initial denaturation at 95°C for 5 min, followed by 15 cycles at 95°C for 1 min, 50°C for 1 min and 72°C for 1 min, with a final extension at 72°C for 7 min. The PCR products from the first step PCR were purified through VAHTSTM DNA Clean Beads. A second round PCR was then performed in a 40 μl reaction which contained 20 μl 2 × Phμsion HF MM, 8 μl ddH2O, 10 μM of each primer and 10 μl PCR products from the first step. Thermal cycling conditions were as follows: an initial denaturation at 98°C for 30 s, followed by 10 cycles at 98°C for 10 s, 65°C for 30 s min and 72°C for 30 s, with a final extension at 72°C for 5 min. Finally, all PCR products were quantified by Quant-iT dsDNA HS Reagent and pooled together. Then, the PCR products were purified with a Qiagen Gel Extraction Kit (Qiagen, Germany). The samples were sequenced on an Illumina NovaSeq platform (Illumina, California, United States), and 250 bp paired-end reads were generated.

### Sequencing Data Analysis

After Illumina NovaSeq sequencing, we obtained paired-end reads, which were merged using FLASH (V1.2.7)^1^ (Magoč and Salzberg, 2011). Then, quality filtering of raw tags was performed to obtain high-quality tag data (clean tags) under strict filtering conditions according to QIIME (V1.9.1)^2^ (Bokulich et al., 2013). The clean tags obtained were further filtered to detect the chimera sequence by UCHIME software. Next, we clustered all the effective tags, and those for which similarity >97% were grouped as operational taxonomic units (OTUs). The Silva database^3^ (Quast et al., 2013) was used based on the Mothur algorithm to annotate taxonomic information. We have uploaded the sequencing data to the NCBI for general scientific community access. The microbial alpha and beta diversities in our samples were calculated in QIIME software and displayed in R software. Alpha diversity was applied to analyze the complexity of species diversity through indexes, including the Chao1 index and Shannon index. Rank sum test analysis was applied to analyze significant differences in alpha diversity. Beta diversity analysis, which was used to evaluate differences in species complexity among the samples, was performed with weighted and unweighted UniFrac distances in QIIME software. Principal coordinates analysis (PCoA) was performed with the stats R package to visualize the distance matrix among all the samples. AMOVA and ADONIS analyses were used to assess significant differences in beta diversity among the three groups.

Linear discriminant analysis (LDA) coupled with effect size (LEfSe) was performed with the LEfSe tool (Hess et al., 2011), and the *p* value was determined by Metastats analysis with the stats R package to discriminate bacterial taxa with significantly different abundances. Only colonies that showed a *P* value <0.05 and a log LDA score >2 were included. A *P* value <0.05 was considered significant.

### Liquid Chromatography Mass Spectrometry/Mass Spectrometry Analysis

The ultra-high performance liquid chromatography coupled with mass spectrometry detection (UHPLC-MS) was applied in our research for the composition of metabolites in the gut and was performed by Shanghai Biotree Biomedical Technology Co., Ltd, China. Fifty milligrams of stool from each sample were weighed in an Eppendorf (EP) tube and then mixed with 1,000 μL of extraction solution [acetonitrile:methanol:water = 2:2:1 (V/V/V)] containing an isotope-labeled internal standard mixture. After 30 s of vortexing, all the samples were homogenized at 35 Hz for 4 min and then sonicated for 5 min in an ice-water bath. After that, the samples were centrifuged at 12,000 rpm for 15 min at 4°C. The resulting supernatant was transferred to a fresh glass vial for analysis. We collected the same amount of supernatant from all samples and prepared QC samples. Untargeted fecal metabolomics analysis was performed with an UHPLC system (Vanquish, Thermo Fisher Scientific) with a UPLC BEH Amide column (2.1 mm × 100 mm, 1.7 μm) coupled to a Q Exactive HFX mass spectrometer (Orbitrap MS, Thermo). The mobile phase consisted of 25 mmol/L ammonium acetate and 25 mmol/L ammonia hydroxide in water (pH = 9.75) (A) and acetonitrile (B). The elution gradient was set as follows: 0∼0.5 min, 95% B; 0.5∼7.0 min, 95%∼65% B; 7.0∼8.0 min, 65%∼40% B; 8.0∼9.0 min, 40% B; 9.0∼9.1 min, 40%∼95% B; and 9.1∼12.0 min, 95% B. The column temperature was 30°*C*. The autosampler temperature was 4°C, and the injection volume was 3 μL. All MS1 and MS2 data were obtained with acquisition software (Xcalibur, Thermo).

### Data Analysis

The raw data was transformed to mzXML format using ProteoWizard and processed by XCMS for peak detection, extraction, alignment, and integration (Smith et al., 2006). Then, we applied an in-house MS2 database for metabolite annotation. Individual peaks were filtered to remove noise by filtering the deviation value using the relative standard deviation method. Subsequently, the missing values missing up to the minimum value were simulated in the raw data. Finally, 4233 peaks remained after the data were processed by the internal standard normalization method. To obtain high-dimensional metabolomic datasets, the final dataset was imported into the SIMCA16.0.2 software package (Sartorius Stedim Data Analytics AB, Umea, Sweden) for principal component analysis (PCA) and orthogonal partial least square discriminant analysis (OPLS-DA) after logarithmic transformation and Pareto scaling. In addition to multivariate statistical methods, Student’s *t*-test was used to identify the altered metabolites in DR patients at univariate level. Metabolites with a variable importance in projection (VIP) value >1 in OPLS-DA analysis and *P* < 0.05 in univariate analysis were considered altered metabolites. In addition, the differential metabolites were mapped into their biochemical pathways through metabolic pathway enrichment and pathway analysis based on MetaboAnalyst 5.0^4^, which uses the high-quality Kyoto Encyclopedia of Genes and Genomes metabolic pathways as the backend knowledge base (Liu et al., 2019; Wang T. et al., 2020). All raw data has been uploaded to NCBI (SUB9930154) and MetaboLights website (MTBLS3012).

### Statistical Analysis

The levels of fecal metabolites and the relative abundances of genera were calculated using Spearman correlation analysis to obtain the corresponding correlation coefficient (Corr) matrix and correlation *P* value matrix. We determined the correlation between only those genera and metabolites for which *P* < 0.05. In all statistical tests, *P* < 0.05 was considered significant.

## Results

### Participant Characteristics

None of the statistics presented in Table 1 for participants recruited in this study was considered significant including hypertension, body mass index (BMI), diabetes duration, glycosylated hemoglobin (HbA1c), total cholesterol, triglyceride or estimated glomerular filtration rate.

**TABLE 1:** Demographic and clinical characteristics of DR patients, DM patients, and healthy controls.

**Characteristic**	**DR patients**	**Healthy control**	**DM patients**	**Total**	**F/H//z/χ^2^**	***P* value**	**Power**
Patient number (*n*)	21	15	14	50	–	–	–
Age (years)	59.57 ± 9.09	56.13 ± 8.88	61.93 ± 6.20	59.20 ± 8.46	1.790^F^	0.178	0.343
Gender (F/M)	7/14	8/7	6/8	21/29	1.443^χ2^	0.486	0.108
BMI (kg/m^2^)	22.79 ± 2.43	21.23 ± 2.07	22.20 ± 1.65	22.16 ± 2.19	2.326^F^	0.109	0.431
Diastolic BP (mm Hg)	133.95 ± 18.15	120.6 ± 14.64	130.29 ± 15.97	129.00 ± 17.14	2.930^F^	0.065	0.542
Systolic BP (mm Hg)	82.24 ± 11.86	75.73 ± 6.03	79.79 ± 8.55	79.6 ± 9.73	2.041^F^	0.141	0.376
Glycated hemoglobin (HbA1c%)	6.44 ± 0.92	5.79 ± 1.14	6.55 ± 1.19	6.29 ± 1.10	2.301^F^	0.111	0.44
Total cholesterol (mmol/L)	4.4 (3.43, 5.04)	3.8 (3.24, 4.51)	4.36 (3.66, 4.36)	4.33 (3.4, 4.9)	2.929^H^	0.231	0.275
Low density lipoprotein (mmol/L)	2.34 ± 0.8	2.48 ± 0.71	2.91 ± 0.62	2.56 ± 0.75	3.069^F^	0.055	0.5
High density lipoprotein (mmol/L)	1.21 ± 0.26	1.37 ± 0.35	1.25 ± 0.22	1.27 ± 0.28	1.600^F^	0.212	0.301
Triglyceride (mmol/L)	1.29 (0.94, 1.73)	1.24 (0.87, 1.34)	1.43 (0.96, 2.08)	1.27 (0.93, 1.58)	2.484^H^	0.289	0.273
Duration of diabetes (years)	13 (5, 19.5)	/	11.5 (2.75, 16.25)	5.5 (0, 15)	−0.76^z^	0.447	0.169
Estimated glomerular filtration rate (ml/min)	98.00 ± 14.65	99.97 ± 8.65	95.67 ± 14.18	97.81 ± 12.94	0.435^F^	0.65	0.134

*The superscript F denotes the F statistic of one-way analysis of variance.*

*^χ2^Analyzed by χ^2^ statistic of chi-square test.*

*^H^Analyzed by the statistic of non-parametric Kruskal-Wallis test.*

*^Z^Analyzed by the statistic of non-parametric Mann-Whitney test.*

*The results of multiple comparisons between groups at the 0.05 level are marked using lowercase letters (abc), the same letter indicates the difference between the two groups is not significant (*p* > 0.05), and different letters indicate that the difference between the two groups is significant (*p* < 0.05). Using pwr.f2.test() in the pwr package (Champely, 2018) in R (Chang and Kwon, 2020; R Development Core Team, 2020).*

### Gut Microbiota Alterations in Patients With Diabetic Retinopathy, Diabetes Mellitus Patients Without Diabetic Retinopathy, and Controls

**FIGURE 1:**
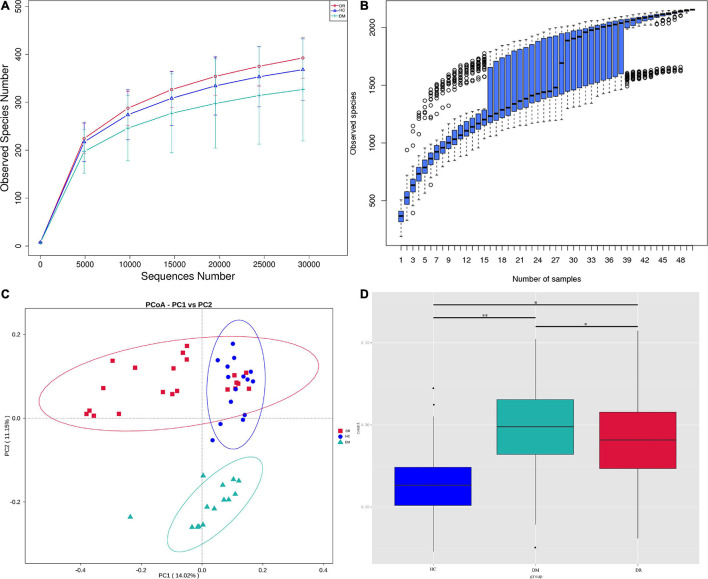
**(A)** Reflectance curve based on OTU count in healthy control group, DR patients and DM patients. DR, DR patients (orange); HC, healthy people (blue); DM, DM patients (green). **(B)** The horizontal coordinate is the sample size; the vertical coordinate is the number of OTUs after sampling. **(C)** The PCoA ordination of Bray-Curtis distances among DR patients, DM patients and healthy controls from 16S rDNA sequencing data. The first three axes of PCoA showed a clear separation. **(D)** Beta diversity between-group difference box plots are based on weighted unifrac distances for the multi-group non-parametric wilcox test. ^∗^*p* < 0.05, ^∗∗^*p* < 0.01.

A total of 2,638,100 effective tags were obtained from the fecal samples of 21 patients with DR, 14 patients with DM and 15 healthy controls, with a mean of 52,762 per sample (ranging from 32,140 to 69,867). The sequences were clustered into OTUs with 97% identity, yielding a total of 2,226 OTUs, and then the OTU sequences were annotated with the Silva 132 database for species annotation. Based on the rarefaction curve (Figure 1A) and the species accumulation boxplot (Figure 1B), the current sequencing and samples were sufficient to identify taxa. The Shannon indexes observed in all three groups (healthy control, T2DM, and DR) were not significantly different (Supplementary Figure 1C). The OTU and Chao1 indexes observed were significantly different between DR patients and DM patients. In addition, the OTU and Chao1 indexes observed between DM patients and healthy controls were also significantly different according to the Wilcoxon test (Supplementary Figure 1), which suggested that the number of microbial communities in DR patients differed from that in DM patients and normal subjects; however, their diversity was not significantly different. In the beta diversity analysis, the gut microbiota could be distinguished among the three groups by PCoA (Figure 1C), which was significant according to ADONIS and AMOVA analyses (Supplementary Table 1). In comparing the boxplots of beta diversity between the groups, when calculated using weighted UniFrac distance, a difference in the gut microbiota was detected between DR patients and healthy controls (*p* = 0), and further comparison of the gut microbiota between DR patients and DM patients likewise produced statistically significant results (*p* = 0.0183) (Figure 1D).

**FIGURE 2:**
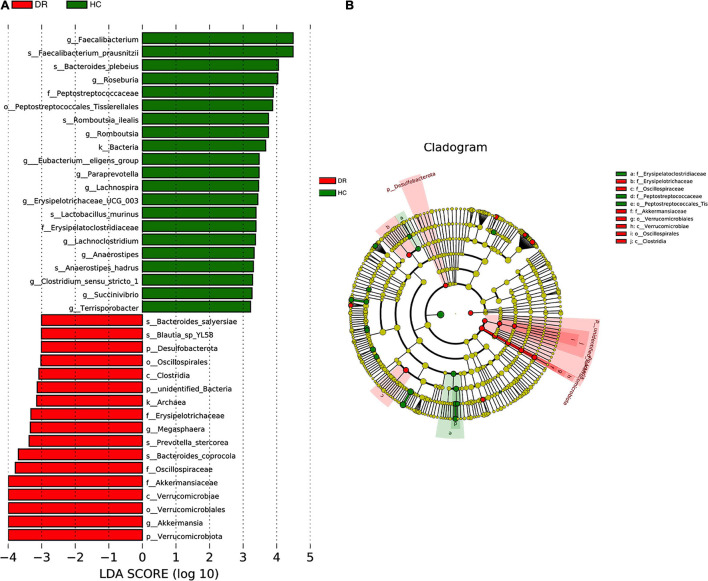
**(A)** Taxa difference between DR patients and healthy controls. LefSe (LDA > 2logs) was used to detect major differences of bacterial taxa between DR patients and normal individuals. 21 bacterial taxa were enriched in healthy controls (green bars) and 17 were enriched in DR patients (red bars). *X*-axis shows the log LDA scores. **(B)** The cladograms of six different taxonomic levels (from kingdom to genus) were constructed. Red circles and shadings show the significantly enriched bacterial taxa were obtained in DR patients. Green circles and shadings show the significantly enriched bacterial taxa obtained in healthy controls.

To determine the differentially abundant bacterial groups in DR patients, we compared them with healthy controls and then performed LEfSe. The results showed that 21 bacterial taxa were enriched in the DR patients, while 17 bacterial taxa were enriched in healthy controls (Figure 2A). Branching maps at six different levels (from kingdom to genus) were obtained by the LEfSe analysis method. The classes Verrucomicrobiae and Clostridia played important roles in the gut microbiota of DR patients. Additionally, not only the orders Verrucomicrobiales and Oscillospirales but also the families Akkermansiaceae and Oscillospiraceae had a greater effect in DR patients (Figure 2B). We also performed lefse analysis between DR and DM and among the three groups (Supplementary Figure 3). Comparison of the relative abundance of microbiota constituents was performed with Metastats analysis and log LDA score, which revealed differences in the gut microbiota between DR patients and healthy controls. The results revealed that at the family level, differences in gut microorganisms existed between DR patients and healthy controls in four families: Oscillospiraceae, Lactobacillaceae, Ruminococcaceae and Lachnospiraceae. At the genus level, *Faecalibacterium*, *Roseburia*, *Lachnospira* and *Romboutsia* were depleted in DR patients compared with healthy controls, and only *Akkermansia* was enriched in DR patients (Supplementary Table 2).

Notably, the use of glucose-lowering drugs, especially metformin, might affect the gut microbiota and could be a confounding factor in this study (Forslund et al., 2015). Based on this possibility, we included all DR and DM patients who received long-term regular oral metformin treatment. To investigate whether the characteristics of the gut microbiota were related just to DR, not to DM, we further compared the composition of the gut microbiota of DR patients with that of DM patients. We discovered that compared to DM patients, DR patients had elevated *Prevotella*, *Faecalibacterium*, *Subdoligranulum*, *Agathobacteria*, and *Olsenella* and reduced *Bacillus*, *Veillonella*, and *Pantoea* abundances at the genus level (Supplementary Table 3). Moreover, we found that *Faecalibacterium* and *Lachnospira* were depleted in DM patients compared with healthy controls at the genus level, and *Klebsiella* and *Enterococcus* were enriched (Supplementary Table 4), which was consistent with Zhao’s study (Zhao et al., 2020).

### Metabolic Alterations in Patients With Diabetic Retinopathy, Diabetes Mellitus Patients Without Diabetic Retinopathy, and Controls

**FIGURE 3:**
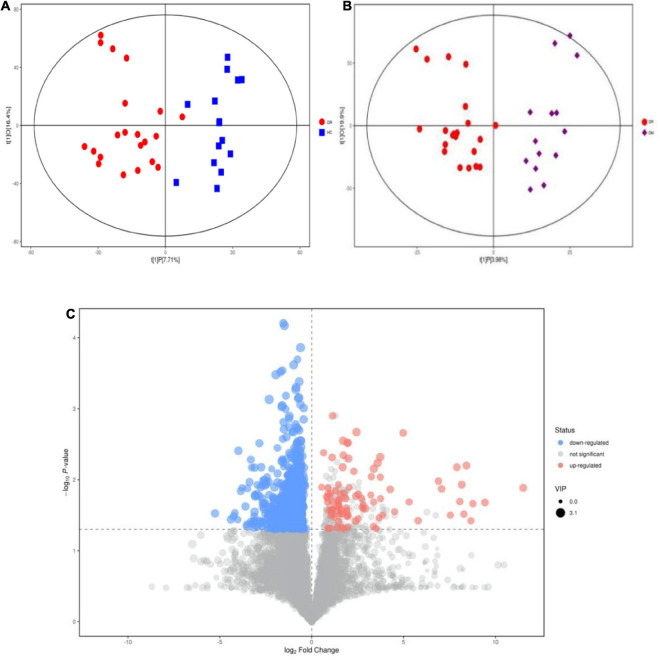
Alteration of metabolites Changes between DR patients and healthy people and between DR patients and DM patients. **(A)** OPLS-DA score samples of fecal samples from DR patients (red circle) and healthy controls (blue square). **(B)** OPLS-DA of fecal samples from DR patients (blue circle) and DM patients (purple square). **(C)** The variation tendencies of fecal metabolites between DR patients and healthy people. The red circles indicate the up-regulated metabolites and blue circles indicate the down-regulated metabolites.

Many studies on the metabolomics of blood and intraocular fluid from DR patients have been conducted (Chen et al., 2016; Haines et al., 2018; Jin et al., 2019). However, stool samples from DR patients have rarely been studied. Hence, we performed a metabolomic analysis of stool samples to discover metabolomic changes in patients with DR. In the OPLS-DA model, significant differences were found in metabolic phenotypes among DR patients, DM patients and healthy controls, suggesting that DR patients may have a unique metabolic profile (Figures 3A,B). The model between DR patients and healthy controls proved to be differential after randomization (*n* = 200) (Supplementary Figure 2A). However, the validity of the model between DR patients and DM patients disappeared after verification (Supplementary Figure 2B), which might mean that the metabolite differences between the two were not significant. As DR was one of the common complications of DM, the two diseases were closely related, which could explain the above results. A volcano map was drawn to depict trends in differentially abundant metabolites (Figure 3C). Four enriched metabolites in DR patients with *p* < 0.05, VIP > 1 and FC (fold change) <0.5 were considered differentially abundant when compared to healthy controls. In addition, 42 metabolites were depleted in the DR samples. By comparing DM patients with healthy controls, seven enriched metabolites and 35 depleted metabolites were found (Supplementary Table).

To further determine the metabolic phenotype of DR, we compared the metabolites between DR patients and DM patients. We found that DR patients had significantly decreased levels of traumatic acid, thromboxane B3, salicyluric acid, pyro-L-glutaminyl-L-glutamine, harman, flazine, butylparaben, betonicin, and β-carboline and increased levels of *N*-gamma-L-glutamyl-D-alanine, *N*-acetyl-L-methionine, L-threo-3-phenylserine, D-proline, armillaramide, and (R)-pelletierine in fecal samples (Table 2).

**TABLE 2:** Identified differential Fecal metabolites between DR patients and DM patients.

**Metabolites**	**Mean DR**	**Mean DM**	**VIP**	***P*-value^a^**	**FC**
Traumatic acid	1.9968E-05	2.59259E-06	1.73	0.025	7.70
Thromboxane B3	1.60099E-05	3.2986E-06	2.06	0.030	4.85
Salicyluric acid	2.52096E-05	9.68573E-06	2.47	0.014	2.60
Pyro-L-glutaminyl-L-glutamine	6.43769E-06	2.58308E-06	1.18	0.032	2.49
*N*-gamma-L-Glutamyl-D-alanine	2.03928E-05	3.82827E-05	2.64	0.007	0.53
*N*-Acetyl-L-methionine	1.36203E-05	3.75546E-05	3.47	0.019	0.36
L-Threo-3-Phenylserine	0.000166407	0.000297864	2.97	0.017	0.56
Harman	0.00061641	0.000132657	1.57	0.003	4.65
Flazine	3.60815E-05	1.54617E-05	1.49	0.033	2.33
D-Proline	2.51415E-05	3.99343E-05	2.58	0.010	0.63
Butylparaben	0.000642486	0.000151561	1.47	0.044	4.24
Betonicine	3.06462E-05	1.10492E-05	1.19	0.045	2.77
Beta-Carboline	0.00031619	0.000119	1.67	0.021	2.66
Armillaramide	1.80033E-05	3.58778E-05	1.96	0.003	0.50
(R)-Pelletierine	6.98284E-06	1.10485E-05	2.05	0.048	0.63

*VIP, variable importance in the projection; FC, fold change. ^a^*P*-value was calculated by Student’s *t*-test.*

**FIGURE 4:**
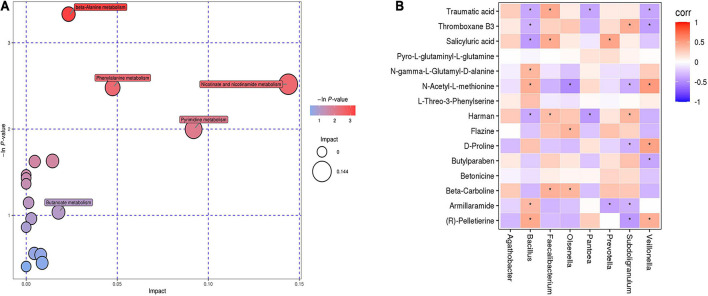
**(A)** Bubble chart of differential metabolic pathways between DR patients and healthy controls. Each bubble in the bubble chart represents a metabolic pathway. **(B)** The relationship between gut microbiota and fecal metabolites in patients with DR. Red indicates that the flora is positively correlated with metabolites, and purple indicates that the flora is negatively correlated with metabolites. “*” indicates a significant difference between the two groups (*p*-value < 0.05).

To identify the metabolic pathways involved in DR, we conducted KEGG annotation and combined the results of powerful pathway enrichment analysis with topological analysis. Metabolic pathway analysis identified 17 pathways with differentially abundant metabolites in DR patients compared with those in the healthy population. The results of the metabolic pathway analysis are shown in bubble plots, revealing β-alanine metabolism, phenylalanine metabolism and nicotinamide metabolism (Figure 4A). In addition, arginine-proline metabolism and α-linolenic acid metabolism pathways showed differentially abundant metabolites between DR and DM patients as detected by KEGG.

### Correlation of the Gut Microbiota and Metabolic Phenotype in Diabetic Retinopathy Patients

To investigate whether gut microbiota composition was associated with the fecal metabolic phenotype in patients with DR, Spearman correlation was performed between the DR and DM groups (Figure 4B). The results showed that *Prevotella* was negatively correlated with armillaramide (*p* = 0.02, *r* = −0.39). *Subdoligranulum* was positively correlated with thromboxane B3 (*p* = 0.01, *r* = 0.42) but negatively correlated with armillaramide (*p* = 0.046, *r* = −0.33). *Bacillus* was positively correlated with armillaramide (*p* = 0.0294, *r* = 0.36) but negatively correlated with traumatic acid (*p* = 0.0248, *r* = −0.37).

## Discussion

In the present study, we identified a distinctive gut microbiota profile in DR patients. Although the gut microbiota alpha diversity and richness analyses did not show significant differences between DR patients and controls, the structure of the microbiome of DR patients changed significantly according to the beta diversity analysis. This research demonstrated dysbiosis of the gut microbiomes in people with DM and DR compared to those in healthy controls. Compared to healthy controls, *Akkermansiaceae* was enriched significantly at the genus level, while *Faecalibacterium* and *Roseburia* were depleted significantly in DR patients. The intestinal bacterium *Akkermansia muciniphila* (an *Akkermansia* species) can specifically degrade mucin (Derrien et al., 2017). Previous studies have suggested that sugar-fed mice have enriched *A. muciniphila* content in the intestine and reduced inner mucus layer thickness, disrupting the intestinal barrier and triggering an inflammatory response (Khan et al., 2020). *A. muciniphila* has been reported to improve glucose tolerance (Greer et al., 2016), prevent fatty liver development and maintain intestinal homeostasis (Kim et al., 2020); however, many studies have confirmed that *A. muciniphila* erodes the intestinal mucus barrier, which could contribute to colitis progression (Desai et al., 2016; Seregin et al., 2017). The significant reduction in the abundances of *Faecalibacterium* spp. and *Roseburia* spp. in the intestines of DR patients is consistent with findings in ulcerative colitis patients (Machiels et al., 2014). Butyrate, which produced by *Faecalibacterium* spp. and *Roseburia* spp., has been shown to block IL-6-induced signal transduction (Yuan et al., 2004). Serving as a vital inflammation pathway, IL-6/STAT3 signaling pathway had been reported to be activated to trigger inflammatory response of the body in ulcerative colitis (Mitsuyama et al., 1995). Similarly, another research had proved that the activation of the NDRG2/IL-6/STAT3 signaling pathway had credible correlation with the development of DR in rats, and protective effect had been confirmed by inhibiting such an important pathway (Wang Y. et al., 2020). The resemblance may suggest that the reduction in *Faecalibacterium* spp. and *Roseburia* spp. abundances in the intestines of DR patients may cause intestinal pathological changes similar to those found in ulcerative colitis patients.

As DR is one of the important complications of DM, we further compared the gut microbiota of DR and DM patients. Compared with DM patients, DR patients exhibited enrichment of gut microbiota constituents such as *Prevotella* and *Subdoligranulum* at the genus level. The genus *Prevotella* is one of the three representative bacteria of the human gut microbiota and is also one of the core genera of human gut microbes (Arumugam et al., 2011; Costea et al., 2018). Previous studies have confirmed a credible correlation of *Prevotella* with inflammatory diseases. *Prevotella* primarily activates Toll-like receptor 2, which can lead to the production of Th17 inflammatory cytokines (Larsen, 2017). IL-17A was originally derived from Th17 cell lineage which was a subtype of CD4 + T cells (Matsuzaki and Umemura, 2018). Studies have confirmed that IL-17A contributed to the development of DR through the IL-17R-Act1-Fas-activated death domain(FADD)axis, which caused endothelial cell death and capillary degeneration in the retina of diabetic patients (Lindstrom et al., 2019). Consequently, we inferred the occurrence of DR was associated with the interaction between *Prevotella* and IL-17R-Act1-Fas-activated death domain axis, further experimental studies are needed to confirm this hypothesis. *Subdoligranulum* is a strictly anaerobic, non-spore-forming gram-negative bacterium that has been shown to be associated with poor metabolism and chronic inflammation, which also lead to disturbances in host metabolism (Yu et al., 2020). *Faecalibacterium*, which has previously been confirmed to have anti-inflammatory effects (Xu et al., 2020), was found to be depleted in DR patients in our study. We speculated that the lack of *Faecalibacterium* could aggravate DR development. At present, there is no direct evidence that *Subdoligranulum* and *Faecalibacterium* is related to the pathogenesis of DR. Likewise, we speculated that the dysregulation of *Subdoligranulum* and *Faecalibacterium* could lead to DR through immune mechanism. Further research could consider to verify our hypothesis.

The above results highlight the potential association of the gut microbiota with DR. However, the altered gut microbiota and its effects on the metabolic phenotype in the host under DR conditions remain unknown and could be a focal point to interpret the possible mechanisms of DR. Therefore, we aimed to assess the impact of the gut microbiota on the fecal metabolic phenotype in DR patients. We found altered fecal metabolite levels in DR patients. Carnosine was depleted in DR patients compared to healthy controls. Carnosine (β-alanyl-l-histidine) is highly abundant in human muscle and brain tissue (Mahootchi et al., 2020) and has strong antioxidant capacity and chelating effects (Boldyrev et al., 2013). Previous studies have also confirmed that various diseases and dysfunctions are associated with alterations in β-alanine and carnosine metabolism, while supplementation with carnosine may be beneficial in multiple sclerosis, diabetic complications and some age-related and neurological diseases (Kirkland and Meyer-Ficca, 2018; Artioli et al., 2019). The levels of succinate, nicotinic acid and niacinamide were decreased in patients with DR. Succinate is an intermediate of the tricarboxylic acid (TCA) cycle and plays a crucial role in adenosine triphosphate (ATP) generation in mitochondria (Mills and O’Neill, 2014). Some studies have confirmed that abnormal mitochondrial function is closely associated with DR (Dehdashtian et al., 2018), which explains the relationship between DR pathogenesis and energy metabolism. Nicotinic acid (niacin or vitamin B3) is a functional group present in the coenzymes nicotinamide adenine dinucleotide (NAD) and nicotinamide adenine dinucleotide phosphate (NADP),which are important cofactors for most cellular redox reactions (Kirkland and Meyer-Ficca, 2018). NAD + , which serves as a regulator of inflammation by acting through sirtuins, has been suggested to play a crucial role in NLRP3 inflammasome activation (He et al., 2012). A variety of NLRP3 activators can inhibit mitochondrial function and therefore limit NAD + concentrations (Misawa et al., 2013). Dysregulation of the NLRP3 inflammasome could act as a contributing factor to the constellation of tissue insults evident in the diabetic retina (Raman and Matsubara, 2020).

Furthermore, two pathways involved in the differential abundance of metabolites between DR and DM patients were found, namely, arginine-proline metabolism and α-linolenic acid metabolism. D-Proline was significantly lower in DR patients than in DM patients and is involved in the arginine-proline metabolic pathway. Evidence suggested that proline was an important nutrient for the retinal pigment epithelium (RPE),which could promot RPE maturation, regulate glucose metabolism and increase the ability of the RPE to withstand oxidative stress (Yam et al., 2019). Recent evidence suggests that the most important nerve cells (photoreceptors) in the retina and the adjacent retinal pigment epithelium (RPE) play an important role in the development of DR (Tonade and Kern, 2021). Therefore, we hypothesize that a decrease in proline content may lead to RPE impairment and thus to the development of DR. Notably, traumatic acid is enriched during α-linolenic acid metabolism. Traumatic acid, which is regarded as an oxidative derivative of unsaturated fatty acids, has been considered to enhance caspase 7 activity, membrane lipid peroxidation and reactive oxygen species (ROS) levels and to play a crucial role in growth and development (Jabłońska-Trypuć et al., 2019). ROS can be maintained in equilibrium and participate in redox reactions in the body. However, when the balance is disturbed, ROS produce retinal cell damage through interactions with cellular components, which could lead to DR development (Calderon et al., 2017). In addition, in the correlation analysis, we found that Bacillus was negatively correlated with traumatic acid. Therefore, we speculate that the decrease in Bacillus abundance leads to an increase in traumatic acid levels, thus leading to DR progression.

We also discovered that armillaramide was negatively correlated with *Prevotella* and *Subdoligranulum* but positively correlated with *Bacillus*. Armillaramide is a lipid-like molecule (Gao et al., 2001) that plays a vital role in maintaining the stability of the membrane structure and is also involved in apoptosis and lipid metabolism pathways (Pruett et al., 2008). However, the exact mechanism of armillaramide in DR is unclear, and we inferred that dysregulation of the intestinal flora could lead to changes in armillaramide content, which contribute to DR occurrence. Further studies are needed to elucidate the specific role of gut dysbiosis and metabolites in the pathogenesis of DR. For instance, we can colonize *Prevotella* and *Subdoligranulum* in the intestine of mice to verify the exact mechanism involved in pathway. We can also conduct targeted analysis on differential metabolites to clarify the relevant role in the pathogenesis.

It is worth noting that, although the permutation test of OPLS-DA model overfit between DR and DM was mentioned above, this was not the only criterion used to determine whether the differences were existed between DR and DM. The student-t test has been widely used in previous studies on metabolomics (Huang et al., 2018; He et al., 2020). When the Student-t test was used to compare metabolites between the DR-DM groups, 15 metabolites were found to be different between the groups. In the future, more evidence should be obtained to validate the accuracy of the differential metabolites by expanding the sample size and performing targeted metabolomics analysis.

To the best of our knowledge, few studies concerning the gut microbiota profile and the analysis of fecal metabolites and their correlation in DR patients have been performed. Plentiful evidence suggests a link between the gut microbiome and hypertension (Li et al., 2017; Yan et al., 2017). A study previously confirmed that gut microbiota-dependent metabolites of trimethylamine n-oxide (tMaO) and its nutrient precursors (choline and l-carnitine) could improve insulin sensitivity during a weight-loss intervention for obese patients (Heianza et al., 2019). Hermes et al. discovered that some gut microbiota patterns were associated with tissue-specific insulin sensitivity in overweight and obese males (Hermes et al., 2020). Hence, we tried to avoid the influence of these confounding factors on the results when selecting individuals. Meanwhile, the demographic and clinical characteristics were simultaneously kept consistent to remove confounding variables such as diabetes duration. Undeniably, some limitations remain in our study. We used R packages to perform power analysis in Table 1. As shown in Table 1, the study was statically underpowered, and a larger sample size will be required in future studies. The heterogeneity of genetic factors, environmental factors, dietary habits, antibiotics regimen, age, sex and ethnicity may lead to changes in the gut microbiota, which could influence the results of our study. Confounding factors could be eliminated by stratified analysis. Of the 50 fecal samples we collected, 48 had been free of antibiotics for more than 3 months in our research. In order to expedite the collection of samples, we selected two samples that had not been on antibiotics for 1–3 months, whose selection criteria also refer to certain literature (He et al., 2020). However, in order to eliminate the interference of antibiotics on the research results, samples free of antibiotics for more than 3 months, even 6 months should be selected in future studies. Research on the exact mechanisms of the gut microbiota and metabolites in DR patients is also needed, which may help provide new ideas for potential treatment.

## Data Availability Statement

The datasets presented in this study can be found in online repositories. The names of the repository/repositories and accession number(s) can be found below: MataboLights accession: MTBLS3012, BioProject accession: PRJNA743182.

## Ethics Statement

The studies involving human participants were reviewed and approved by The Ethics Committee of the Second Affiliated Hospital of Chongqing Medical University. The patients/participants provided their written informed consent to participate in this study. Written informed consent was obtained from the individual(s) for the publication of any potentially identifiable images or data included in this article.

## Author Contributions

MZ and ZZo conceived the idea and designed the experiments. ZZo, MZ, and ZZe collected the sample, analyzed the data, and wrote the manuscript. XX, XC, JnP, HY, and JaP interpreted data and revised the manuscript. All authors contributed to the article and approved the submitted version.

## Conflict of Interest

The authors declare that the research was conducted in the absence of any commercial or financial relationships that could be construed as a potential conflict of interest.

## Publisher’s Note

All claims expressed in this article are solely those of the authors and do not necessarily represent those of their affiliated organizations, or those of the publisher, the editors and the reviewers. Any product that may be evaluated in this article, or claim that may be made by its manufacturer, is not guaranteed or endorsed by the publisher.

## Footnotes

1
http://ccb.jhu.edu/software/FLASH/

2
http://qiime.org/scripts/split_libraries_fastq.html

3
http://www.arb-silva.de/

4
http://www.metaboanalyst.ca
