# Supplementary material for: Gut Microbiota Composition and Fecal Metabolic Profiling in Patients With Diabetic Retinopathy
Source: Front Cell Dev Biol. 2021 Oct 15;9:732204. doi: 10.3389/fcell.2021.732204 (PMC8554156; doi:10.3389/fcell.2021.732204)
Supplement: Supplementary file 2 [file Data_Sheet_1.PDF]

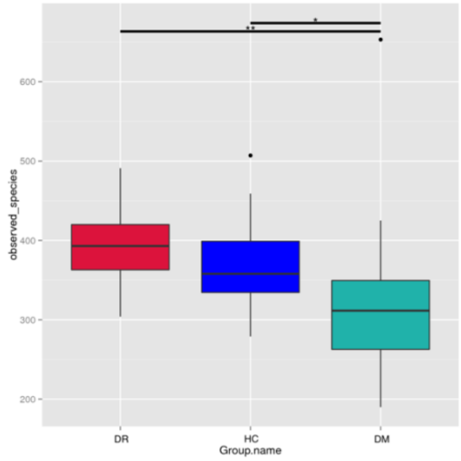

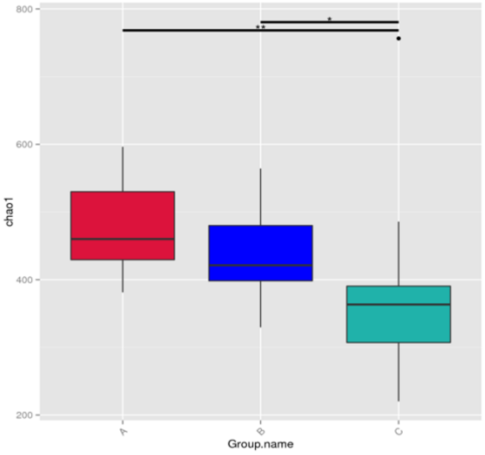

A

B

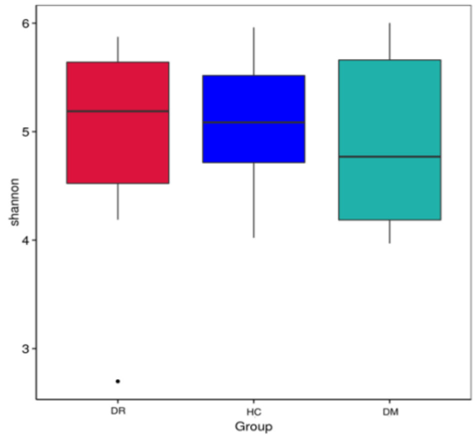

C

Fig.S1: (A, B) Alpha diversity between-group are based on the OTU and Chao1 indices for wilcox test. *p < 0.05, **p < 0.01. (C) Shannon index has no significant difference between the DR-DM, HC-DM, and DR-HC groups (p> 0.05), and the p values are 0.4280, 0.4369, 0.9637.

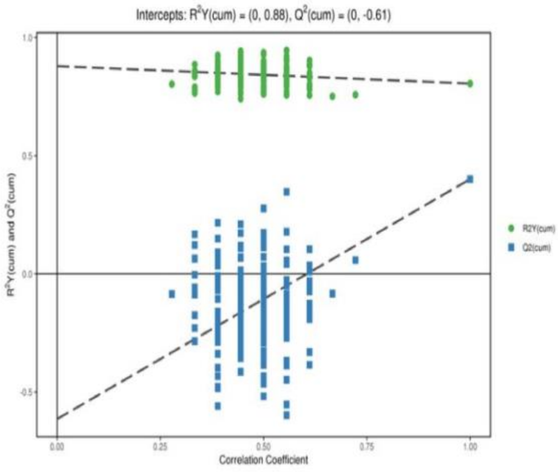

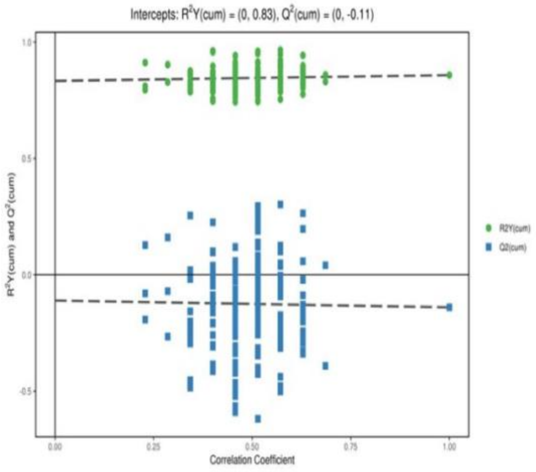

A

B

FigS2. (A) Validation of OPLS-DA model between DR patients and healthy people (using 200 random permutations). (B) Validation of OPLS-DA model between DR patients and DM patients (using 200 random permutations).

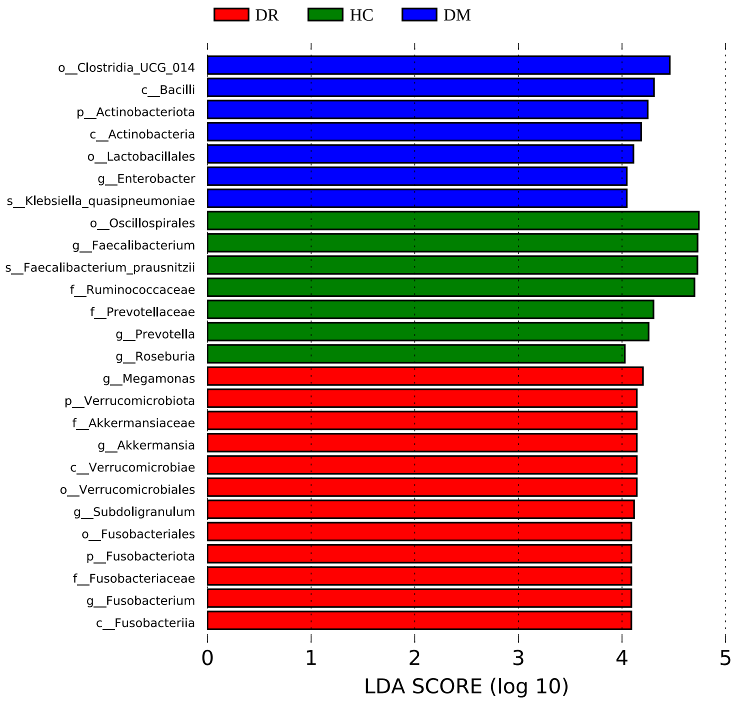

A

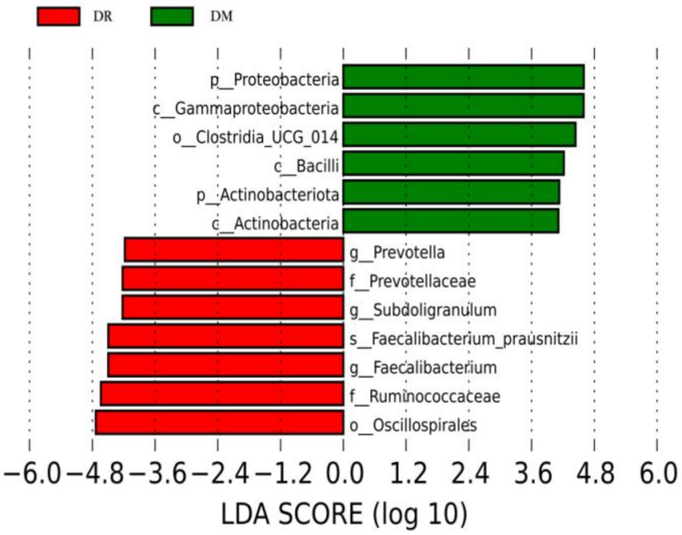

B

FigS3. (A) Taxa difference among DR patients, DM patients and healthy controls. (B) Taxa difference between DR patients and DM patients. LefSe analysis was used to detect major differences of bacterial taxa.

TableS1: ADONIS and AMOVA analysis are based on weighted or unweighted Unifrac distance matrix analysis of different groups.

bray_adonis						
Vs_group	Df	SumsOfSqs	MeanSqs	F.Model	R2	Pr(>F)
DR-HC	1(34)	0.3987(7.5566)	0.39867(0.22225)	1.7938	0.05011(0.94989)	0.01
DR-DM	1(33)	0.7289(8.4088)	0.72889(0.25481)	2.8605	0.07977(0.92023)	0.001
HC-DM	1(27)	0.9043(5.9167)	0.90427(0.21914)	4.1265	0.13257(0.86743)	0.001
unweighted_unifrac_amova						
vs_group	SS	df	MS	Fs	p-value	
DR-HC-DM	1.35247(7.14495)	2(47)	0.676233(0.15202)	4.4483	<0.001	
DR-HC	0.496952(4.86878)	1(34)	0.496952(0.143199)	3.4704	<0.001	
HC-DM	0.698351(3.80519)	1(27)	0.698351(0.140933)	4.9552	<0.001	

TableS2: List of significantly different gut microbiota between DR patients and HC patients. For each taxonomical group, the average relative abundance and the P-value of the metastats analysis are reported. Only P-values<0.05 are shown.

Phylogenetic level	Taxonomy	log LDA scores	DR Avg rel. ab	DH Avg rel. ab	P-value[a]
Class	Clostridia	3.60	0.0039	0.0013	9E-04
Order	Oscillospirales	3.556	0.0036	0.0012	0.002
Family	Oscillospiraceae	4.406	0.0255	0.0177	0.003
	Lactobacillaceae	4.122	0.0002	5.23E-05	0.003
	Ruminococcaceae	3.274	0.0018	0.0003	0.004
	Lachnospiraceae	5.379	0.0002	5.23E-05	0.003
Genus	Faecalibacterium	5.189	0.0959	0.1548	0.023
	Akkermansiaceae	4.30	0.0199	0.0004	0.026
	Roseburia	4.514	0.00085	0.0326	0.003
	Lachnospira	3.903	0.0016	0.008	0.029
	Romboutsia	4.272	0.007	0.0187	0.01

p-values were determined using the student’s test.

TableS3: List of significantly different gut microbiota between DR patients and DM patients. Only P-values<0.05 are shown.

Phylogenetic level	Taxonomy	log LDA scores	DR Avg rel. ab	DM Avg rel. ab	P-value[a]

Genus	Prevotella	4.528	0.0337	0.0025	0.0129
	Faecalibacterium	4.981	0.0959	0.031	0.0019
	Subdoligranulum	4.577	0.037	0.0105	0.0099
	Agathobacter	4.503	0.0381	0.0115	0.0089
	Bacillus	3.993	5.85E-05	0.0098	0.0009
	Veillonella	3.439	0.0001	0.0027	0.0009
	Pantoea	4.501	0.0023	0.0112	0.0039
	Olsenella	3.24	0.0017	3.66E-05	0.0059

p-values were determined using the student's test.

TableS4: List of significantly different gut microbiota between DM patients and HC patients. Only P-values<0.05 are shown.

Phylogenetic level	Taxonomy	log LDA scores	DM Avg rel. ab	HC Avg rel. ab	P-value[a]
Genus	Agathobacter	4.533	0.0115	0.0341	0.0119
	Faecalibacterium	5.189	0.031	0.1548	0.0009
	Roseburia	4.514	0.0136	0.0326	0.0179
	Weissella	3.925	0.0084	0.0003	0.0009
	Bacillus	3.993	0.0098	3.18E-05	0.0009
	Enterococcus	3.25	0.0017	0.0001	0.0009
	Klebsiella	2.601	0.0003	4.78E-05	0.0019
	Lachnospira	3.903	0.0005	0.0005	0.0009

p-values were determined using the student's test.
